# ICOS controls Foxp3^+^ regulatory T-cell expansion, maintenance and IL-10 production during helminth infection

**DOI:** 10.1002/eji.201242794

**Published:** 2013-01-14

**Authors:** Stephen A Redpath, Nienke van der Werf, Ana M Cervera, Andrew S MacDonald, David Gray, Rick M Maizels, Matthew D Taylor

**Affiliations:** 1 Institute of Immunology and Infection Research, School of Biological Sciences, University of EdinburghEdinburgh, UK; 2 Centre for Immunity, Infection and Evolution, School of Biological Sciences, University of EdinburghEdinburgh, UK; 3 Department of Experimental Immunology, Academic Medical Center, University of AmsterdamAmsterdam, The Netherlands; 4 Laboratory of Cellular Signaling, Department of Cardiovascular Development and Repair, Centro Nacional de Investigaciones CardiovascularesMadrid, Spain

**Keywords:** Co-stimulatory molecules, Immune regulation, Parasitology, Regulatory T (Treg) cells, T helper (Th) cells

## Abstract

Foxp3^+^ regulatory T (Treg) cells are key immune regulators during helminth infections, and identifying the mechanisms governing their induction is of principal importance for the design of treatments for helminth infections, allergies and autoimmunity. Little is yet known regarding the co-stimulatory environment that favours the development of Foxp3^+^ Treg-cell responses during helminth infections. As recent evidence implicates the co-stimulatory receptor ICOS in defining Foxp3^+^ Treg-cell functions, we investigated the role of ICOS in helminth-induced Foxp3^+^ Treg-cell responses. Infection of ICOS^−/−^ mice with *Heligmosomoides polygyrus* or *Schistosoma mansoni* led to a reduced expansion and maintenance of Foxp3^+^ Treg cells. Moreover, during *H. polygyrus* infection, ICOS deficiency resulted in increased Foxp3^+^ Treg-cell apoptosis, a Foxp3^+^ Treg-cell specific impairment in IL-10 production, and a failure to mount putatively adaptive Helios^−^Foxp3^+^ Treg-cell responses within the intestinal lamina propria. Impaired lamina propria Foxp3^+^ Treg-cell responses were associated with increased production of IL-4 and IL-13 by CD4^+^ T cells, demonstrating that ICOS dominantly downregulates Type 2 responses at the infection site, sharply contrasting with its Type 2-promoting effects within lymphoid tissue. Thus, ICOS regulates Type 2 immunity in a tissue-specific manner, and plays a key role in driving Foxp3^+^ Treg-cell expansion and function during helminth infections.

## Introduction

Helminth parasites excel at subverting the host's immune regulatory pathways resulting in immunosuppressed hosts harbouring chronic infections 1,2. This immune suppression forms a major barrier to the acquisition of protective Th2 immunity, both in regard to natural infections and potential vaccination. At the same time, immune downregulation plays a beneficial role in protecting the host from pathology during chronic infection, and helminth infections are linked to the amelioration of allergy and auto-immune diseases indicating that helminth-induced immune suppression can be therapeutically applied to the treatment of these conditions 3,4.

Foxp3^+^ regulatory T (Treg) cells play central downregulatory roles in controlling reactivity to self-Ags and preventing auto-immune diseases 5, as well as in limiting inflammatory responses during infection 6,7. Helminths induce dominant Foxp3^+^ Treg-cell responses that inhibit protective immunity 8–11, alleviate immune pathology 12–14 and can protect against allergic inflammation 15. Thus, Foxp3^+^ Treg cells are a fundamental mechanism of immune regulation during helminth infections, and an understanding of the mechanisms governing the induction of Foxp3^+^ Treg-cell responses is of principal importance for the design of both prophylactic helminth treatments and therapies for allergies and autoimmunity.

Alongside its noted roles in promoting Th1, Th2 and Th17 effector T (Teff) cell responses 16, and in particular T follicular helper (Tfh) cells 17, recent evidence indicates that the T-cell co-stimulatory molecule ICOS (CD278) is involved in the development and function of Treg cells. ICOS signalling is required for Foxp3^+^ Treg-cell suppression in autoimmune settings 18–20, and for the expansion of Foxp3^+^ Treg cells following Ag challenge 21. Moreover, ICOS expression is linked to the production of IL-10 by CD4^+^ Teff cells 22, the induction of regulatory Foxp3^−^ Tr1 cells 23–26, and can be used to define a subset of Foxp3^+^CD4^+^ Treg cells that suppresses via IL-10 27. In infections with parasitic helminths, ICOS is important for the development of Th2 and Ab responses towards the nematodes *Nippostrongylus brasiliensis*, *Trichuris muris*, *Trichinella spiralis* and *Brugia malayi* 28–31. Interestingly, alongside its association with CD4^+^ Teff cells, ICOS expression is also upregulated on Foxp3^+^ Treg cells elicited in response to the filarial nematode *Litomosoides sigmodontis* 10. However, the role of ICOS in generating Foxp3^+^ Treg-cell responses towards helminth parasites is unknown.

In this study we tested the hypothesis that ICOS is required for the induction and function of Foxp3^+^ Treg-cell responses during helminth infection. ICOS deficiency led to reduced expansion and maintenance of Foxp3^+^ Treg cells within secondary lymphoid tissue in response to the intestinal nematode *Heligmosomoides polygyrus* and the trematode *Schistosoma mansoni*. Within the lamina propria (LP), *H. polygyrus* elicited Foxp3^+^ Treg cells were all negative for expression of Helios, a putative natural Foxp3^+^ Treg-cell marker 32, and this population was absent in ICOS^−/−^ mice suggesting the induction of an ICOS-dependent adaptive Helios^−^Foxp3^+^ Treg-cell population. Moreover, ICOS^−/−^ mice showed a Foxp3^+^ Treg-cell specific impairment in IL-10 in response to *H. polygyrus*, and increased levels of apoptosis. Interestingly, within the LP the impaired Foxp3^+^ Treg-cell response in ICOS^−/−^ mice associated with an increased proportion of CD4^+^ T cells producing IL-4 and IL-13. This enhancement of Type 2 immunity at the infection site sharply contrasted with the mesenteric LN (MLN), where ICOS deficiency led to weakened Type 2 responses. Thus, ICOS controls Type 2 immunity in a tissue-specific manner, and plays a key role regulating Foxp3^+^ Treg-cell induction, maintenance and function during helminth infection.

## Results

### CD4^+^Foxp3^+^ Treg cells and CD4^+^Foxp3^−^ Teff cells upregulate ICOS upon helminth infection

**Figure 1:**
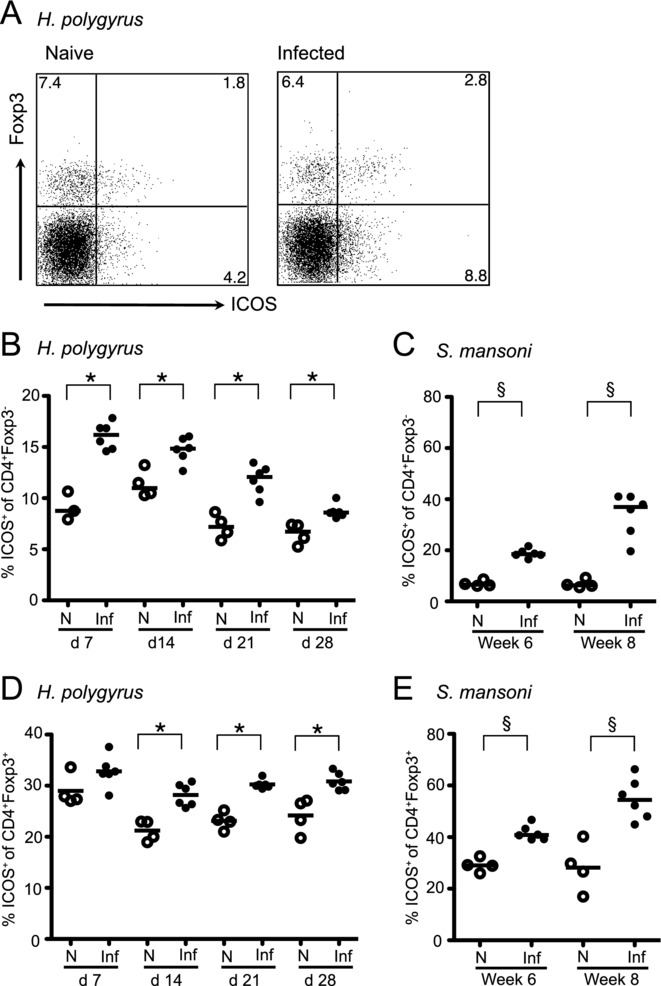
Foxp3^+^ Treg cells and Foxp3^−^ Teff cells increase expression of ICOS in response to helminth infection. C57BL/6 mice were infected with *H. polygyrus* or *S. mansoni* and the expression of ICOS by CD4^+^Foxp3^+^ Treg cells and CD4^+^Foxp3^−^ Teff cells assessed over time. (A) Representative staining for Foxp3 and ICOS on MLN CD4^+^ T cells from naïve and *H. polygyrus*-infected mice 14 day pi. (B, C) Percentage of CD4^+^Foxp3^−^ cells expressing ICOS from (B) the MLNs of *H. polygyrus*-infected mice and (C) spleen of *S. mansoni*-infected mice. (D, E) Percentage of CD4^+^Foxp3^+^ cells expressing ICOS from (D) the MLNs of *H. polygyrus*-infected and (E) spleen of *S. mansoni*-infected mice. Symbols denote individual naïve (open) and infected (closed) mice, and lines denote means (B, D) or medians (C, E). Results show one representative experiment of two performed for each time point. *p < 0.005 (ANOVA using combined data from two separate experiments). ^§^*p* < 0.05 (Mann–Whitney).

We previously observed that CD4^+^Foxp3^+^ Treg cells upregulate ICOS during infection with filarial nematode parasites 10. As ICOS is involved in a range of T-cell responses, we hypothesised that ICOS co-stimulation represents a common pathway by which Foxp3^+^ Treg-cell responses are promoted to distinct helminths. To test this, we infected C57BL/6 mice with the nematode *H. polygyrus*, or with the trematode *S. mansoni*, and determined the expression of ICOS by CD4^+^ T cells in the MLNs and spleen, respectively. Alongside the expected upregulation of ICOS by CD4^+^Foxp3^−^ Teff cells during both infections (Fig. 1A–C), CD4^+^Foxp3^+^ Treg cells showed increased expression of ICOS over the first 4 weeks of *H. polygyrus* infection (Fig. 1D) and during the acute egg phase (weeks 6–8) of *S. mansoni* infection (Fig. 1E). Thus, upregulation of ICOS by Foxp3^+^ Treg cells is a common feature of both nematode and trematode infections.

### ICOS promotes the expansion and maintenance of Foxp3^+^ Treg cells during helminth infection

**Figure 2:**
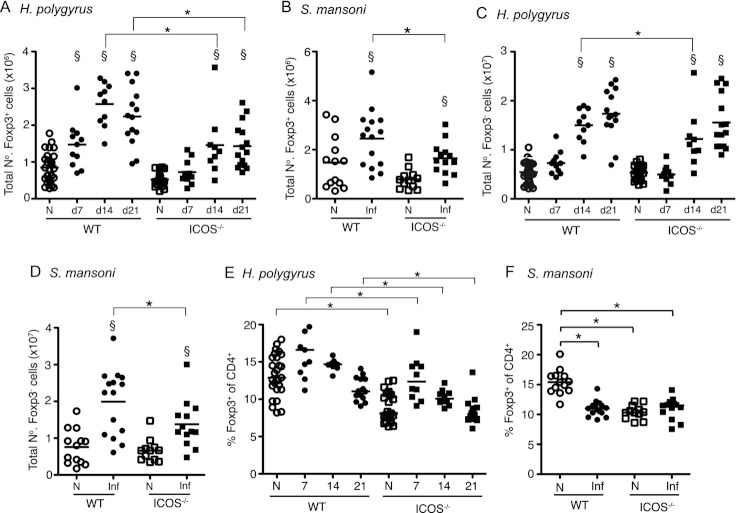
ICOS is required for the expansion and maintenance of CD4^+^Foxp3^+^ Treg cells during *H. polygyrus* and *S. mansoni* infections. The numbers of (A, B) CD4^+^Foxp3^+^ Treg cells, (C, D) numbers of CD4^+^Foxp3^−^ Teff cells, and (E, F) percentages of CD4^+^Foxp3^+^ Treg cells were quantified in WT (circles) and ICOS^−/−^ (squares) C57BL/6 mice (A, C, E) in the MLNs of *H. polygyrus*-infected mice between days 7 and 21, and (B, D, F) in the spleen of *S. mansoni*-infected mice at week 8. Open symbols represent naïve mice and closed symbols represent infected mice. Data shown are pooled from three independent experiments, symbols denote individual mice and lines denote mean values. *Significant strain effect and § significant infection effect (*p* < 0.05, Tukey HSD, ANOVA performed using combined data from two or three separate experiments).

To determine whether ICOS is required for the generation of Foxp3^+^ Treg-cell responses during helminth infection, we infected C57BL/6 ICOS^−/−^ 33 and WT mice with *H. polygyrus* or *S. mansoni*. Upon *H. polygyrus* infection the numbers of Foxp3^+^ Treg cells in the MLN of WT mice significantly increased 73% by day 7 post-infection (pi), however, there was no early expansion of Foxp3^+^ Treg cells at this time point in ICOS^−/−^ mice (Fig. 2A). A delayed increase in Foxp3^+^ Treg cells was observed in the ICOS^−/−^ mice by day 14, but they remained at significantly lower numbers than in WT mice through to day 21 pi. Similarly, WT mice infected with *S. mansoni* had increased numbers of splenic CD4^+^Foxp3^+^ Treg cells at 8 weeks pi, and this increase was significantly lower in ICOS^−/−^ mice (Fig. 2B). Within *H. polygyrus*-infected WT mice the CD4^+^Foxp3^−^ Teff-cell population expanded at a slower rate than the CD4^+^Foxp3^+^ Treg cells, not increasing significantly until day 14 of infection (Fig. 2C). Thus, as with filarial parasites 10, *H. polygyrus* biases the early immune response towards a Treg-cell phenotype. Similar to the CD4^+^Foxp3^+^ Treg-cell population, ICOS^−/−^ mice had significantly reduced numbers of CD4^+^Foxp3^−^ Teff cells during infections with both *H. polygyrus* (Fig. 2C) and *S. mansoni* (Fig. 2D).

As previously reported 21, the percentage of CD4^+^Foxp3^+^ Treg cells within the LN and spleen of naïve ICOS^−/−^ mice was significantly reduced (Fig. 2E and F). Infection with *H. polygyrus* did not change the percentage of MLN CD4^+^Foxp3^+^ Treg cells in either WT or ICOS^−/−^ mice (Fig. 2E), indicating that ICOS deficiency impaired the expansion of CD4^+^Foxp3^+^ Treg cells and CD4^+^Foxp3^−^ Teff cells to a similar extent. *Schistosoma mansoni* infection caused a significant reduction in the percentage of splenic CD4^+^Foxp3^+^ Treg cells in WT, but not ICOS^−/−^, mice at week 8 pi (Fig. 2F). Thus, ICOS deficiency had a greater effect on the expansion of splenic CD4^+^Foxp3^−^ Teff cells than CD4^+^Foxp3^+^ Treg cells at week 8 of *S. mansoni* infection. However, due to the lower basal percentage of splenic CD4^+^Foxp3^+^ Treg cells in ICOS^−/−^ mice, there was no significant difference in percentages between infected WT and ICOS^−/−^ mice. Consistent with ICOS deficiency simultaneously impairing Teff- and Treg-cell responses there was no effect on susceptibility to *H. polygyrus* or *S. mansoni* infections (Supporting Information Fig. 1A–D). Similarly, although Ab mediated blockade of ICOS has been reported to increase granulatomous responses to *S. mansoni* eggs 34, there was no change in the size of egg-induced granulomas during *S. mansoni* infection (Supporting Information Fig. 1E and F). In summary, alongside its role in controlling CD4^+^ Teff-cell responses, ICOS co-stimulation promotes the expansion and maintenance of Foxp3^+^ Treg cells in both nematode and trematode infections.

### ICOS^−/−^ mice fail to generate a Helios^−^Foxp3^+^ Treg-cell response to *H. polygyrus* within the LP

**Figure 3:**
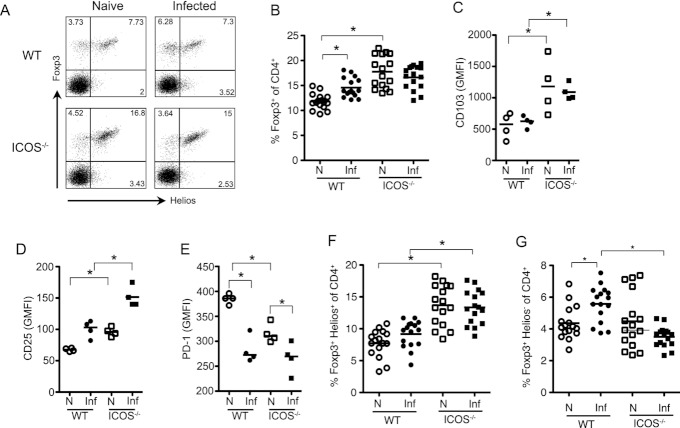
ICOS^−/−^ mice show impaired expansion of Helios^−^Foxp3^+^ Treg cells within the LP. WT (circles) and ICOS^−/−^ (squares) mice were infected with *H. polygyrus* and the expression of Foxp3, CD103, CD25, PD-1 and Helios by LP CD4^+^ T cells assessed at day 7 pi. (A) Representative staining for Foxp3 and Helios on CD4^+^ T cells. (B) Percentage of LP CD4^+^ T cells expressing Foxp3 was determined by flow cytometry. (C–E) Geometric mean expression intensity of (C) CD103, (D) CD25 and (E) PD-1 on CD4^+^Foxp3^+^ Treg cells. (F and G) Percentage of (F) Helios^+^Foxp3^+^ and (G) Helios^−^Foxp3^+^ Treg cells is shown. Data shown are from (B, F, G) four independent experiments or from (C, D, E) one experiment representative of three performed. Open symbols represent naïve mice and closed symbols represent infected WT mice. *p < 0.05 (Tukey HSD, ANOVA performed using combined data from three or four experiments).

Studies on the role of ICOS in T-cell biology have focussed on secondary lymphoid tissue. Therefore, to determine whether ICOS deficiency has a similar impact on Foxp3^+^ Treg cells at the infection site, we compared Foxp3^+^ Treg-cell responses in the LP of the small intestine of *H. polygyrus*-infected WT and ICOS^−/−^ mice. Interestingly, whilst naïve ICOS^−/−^ mice had a lower basal percentage of CD4^+^Foxp3^+^ Treg cells in their MLNs consistent with published work 21 (Fig. 2E and F), the proportion of CD4^+^Foxp3^+^ Treg cells in the LP of uninfected ICOS^−/−^ mice was significantly greater than in WT mice (Fig. 3A and B). This was associated with increased expression of CD103 and CD25 by CD4^+^Foxp3^+^ Treg cells in ICOS^−/−^ mice (Fig. 3C and D), and with reduced expression of the inhibitory receptor PD-1 (Fig. 3E) that can inhibit Foxp3^+^ Treg-cell expansion 35. Following *H. polygyrus* infection the CD4^+^Foxp3^+^ Treg-cell population increased in proportion in WT, but not ICOS^−/−^ mice, by day 7 pi (Fig. 3B). Thus, ICOS^−/−^ mice have a higher basal level of Foxp3^+^ Treg cells within their LP that show a heightened activation phenotype, but that fail to expand upon challenge with *H. polygyrus*.

*Heligmosomoides polygyrus* promotes the generation of adaptive Foxp3^+^ Treg cells 36, and the intestine favours adaptive Foxp3^+^ Treg-cell responses 37. Thus, expression of Helios, a putative marker for natural Foxp3^+^ Treg cells 32, was measured to test whether ICOS deficiency differentially affected natural Helios^+^ versus adaptive Helios^−^ Foxp3^+^ Treg cells. The elevated basal level of LP Foxp3^+^ Treg cells in naïve ICOS^−/−^ mice solely comprised Helios^+^Foxp3^+^ Treg cells indicating expansion of natural Foxp3^+^ Treg cells (Fig. 3F and G). Interestingly, the day 7 LP Foxp3^+^ Treg-cell response elicited by *H. polygyrus* in WT mice was entirely Helios^−^ in nature, suggesting the preferential expansion of adaptive Foxp3^+^ Treg cells. This expansion of adaptive Helios^−^Foxp3^+^ Treg cells by *H. polygyrus* was absent in the ICOS^−/−^ mice, indicating that ICOS^−/−^ mice fail to mount an adaptive LP Helios^−^Foxp3^+^ Treg-cell response during *H. polygyrus* infection (Fig. 3G). In contrast to the LP, *H. polygyrus* significantly expanded both Helios^−^Foxp3^+^ and Helios^+^Foxp3^+^ Treg cells within the MLN of WT mice at day 7 pi, and both populations failed to expand in ICOS^−/−^ mice (Supporting Information Fig. 2). Thus, upon infection, *H. polygyrus* preferentially expands Helios^−^Foxp3^+^ adaptive Treg cells within the LP in an ICOS-dependent manner.

### ICOS downregulates localised Th2 cell responses within the LP

**Figure 4:**
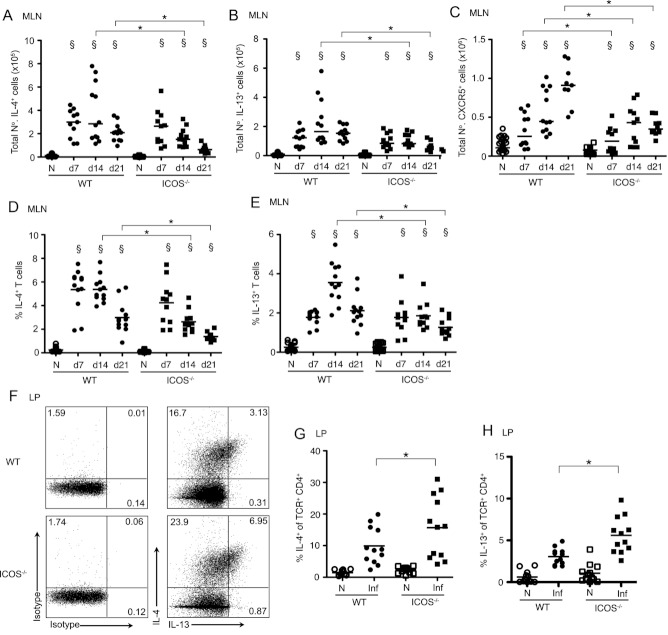
ICOS deficiency results in increased Th2 cytokine production by LP CD4^+^ T cells. WT (circles) and ICOS^−/−^ (squares) mice were infected with *H. polygyrus* and flow cytometry was used to quantify the secretion of IL-4 and IL-13 by MLN and LP CD4^+^ T cells. (A–C) The number of MLN CD4^+^ T cells producing (A) IL-4 and (B) IL-13, and (C) expressing CXCR5. (D, E) The percentage of MLN CD4^+^ T cells producing (D) IL-4 and (E) IL-13. (F) Representative staining for IL-4 and IL-13 on LP CD4^+^ T cells. (G and H) Percentage of LP CD4^+^ T cells producing (G) IL-4 and (H) IL-13. Symbols denote individual naïve (open symbols) and infected (closed symbols) animals and lines represent means. (A–F) Data shown are pooled from three independent experiments. *denotes significant strain effect and § denotes significant infection effect (*p* < 0.05, Tukey HSD, ANOVA performed using combined data from three separate experiments).

ICOS deficiency is associated with reduced Type 2 responses within peripheral LNs 28–30. Recent studies now attribute this to the loss of ICOS-dependent IL-4 secreting Tfh cells, rather than Th2 cells as originally thought 38–40. CD4^+^ T cells do not commit to the Tfh lineage until days 6–10 of infection 39, and at day 7 of *H. polygyrus* infection, on the cusp of Tfh-cell commitment, the numbers of IL-4 and IL-13 producing CD4^+^ T cells in the MLNs were equivalent in WT and ICOS^−/−^ mice (Fig. 4A and B). By day 14 of *H. polygyrus* infection ICOS^−/−^ mice had reduced numbers of CD4^+^ T cells producing IL-4 and IL-13 within the MLNs, which was mirrored by an impaired expansion of MLN CD4^+^CXCR5^+^ Tfh cells (Fig. 4A–C). A similar pattern was observed for the percentage of CD4^+^ T cells producing IL-4 and IL-13 (Fig. 4D and E). Thus, in agreement with other studies 39, ICOS does not appear to be required for the priming of IL-4 and IL-13 secreting CD4^+^ T cells prior to Tfh-cell commitment, and the subsequent loss of Type 2 cytokines appears to be driven by an impaired ability to generate a CD4^+^CXCR5^+^ Tfh-cell response.

As the effect of ICOS deficiency on Type 2 responses within the MLN is dominated by its impact on Tfh cells, to determine the impact of ICOS on Th2 cells in the absence of Tfh cells, we assessed the production of IL-4 and IL-13 by CD4^+^ T cells in the LP. Strikingly, the percentage of CD4^+^ T cells producing these cytokines within the LP at day 7 pi was significantly greater (1.6-fold and 2-fold, respectively) in ICOS^−/−^ compared to WT mice (Fig. 4F–H). Thus, in contrast to the MLN, ICOS deficiency resulted in an elevated production of Type 2 cytokines by CD4^+^ T cells within the LP, suggesting that ICOS downregulates Th2 cells at the infection site.

### ICOS deficiency impairs the proliferation of CD4^+^Foxp3^−^ Teff cells, but not CD4^+^Foxp3^+^ Treg cells

**Figure 5:**
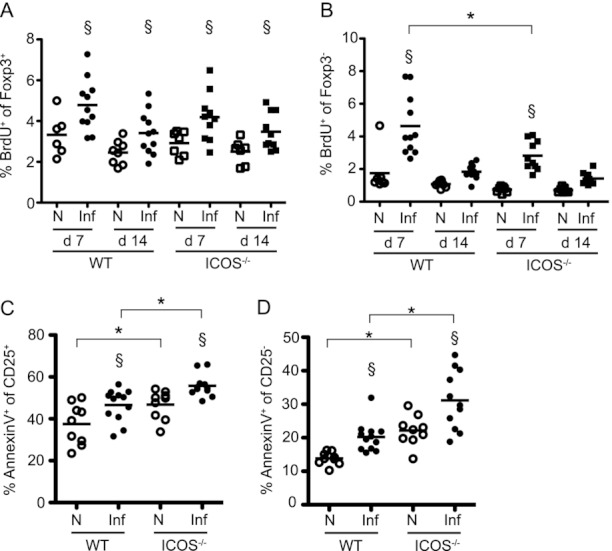
ICOS deficiency increases Treg-cell apoptosis, but not proliferation, during *H. polygyrus* infection. (A, B) WT (circles) and ICOS^−/−^ (squares) mice were infected with *H. polygyrus* and the in vivo uptake of BrdU by (A) CD4^+^Foxp3^+^ Treg cells and (B) CD4^+^Foxp3^−^ Teff cells from the MLN quantified on days 7–21 pi. (C and D) Ex vivo percentage of MLN (C) CD4^+^CD25^+^ Treg cells and (D) CD4^+^CD25^−^ Teff cells positive for Annexin V on day 7 of *H. polygyrus* infection is shown. Data shown are pooled from two (A and B) or three (C and D) independent experiments. Open symbols represent naïve mice and closed symbols represent infected mice, lines denote mean values. Significant effect of strain (*) and infection (§) (*p* < 0.05, Tukey HSD). (A and B) ANOVA was performed using combined data from two experiments. (C and D) ANOVA was performed using combined data from three experiments with experiment as a random variable.

The reduced Foxp3^+^ Treg-cell responses observed in the MLN of ICOS^−/−^ mice could be due to a variety of reasons. To determine whether the failed expansion of CD4^+^Foxp3^+^ Treg cells within ICOS^−/−^ mice was due to impaired proliferation, we labelled dividing cells in vivo by administration of BrdU to *H. polygyrus*-infected WT and ICOS^−/−^ mice 1 day prior to autopsy. The percentage of BrdU^+^Foxp3^+^ cells within the MLN significantly increased in both strains of mice upon infection demonstrating that Foxp3^+^ Treg cells proliferate in response to *H. polygyrus* infection (Fig. 5A). However, there was no difference in BrdU uptake by Foxp3^+^ Treg cells between infected ICOS^−/−^ and WT mice, indicating that ICOS is not required for Foxp3^+^ Treg-cell proliferation and that the ICOS-mediated Foxp3 deficiency is not due to reduced cell division. In contrast, BrdU uptake by CD4^+^Foxp3^−^ Teff cells at day 7 of infection was severely diminished in ICOS^−/−^ mice (Fig. 5B). Although, by day 14 the percentage of BrdU^+^Foxp3^−^ Teff cells in the two strains had equalised indicating that ICOS deficiency only impacts upon Teff-cell proliferation during the early stages of priming. Therefore, ICOS deficiency limits the expansion of CD4^+^Foxp3^+^ Treg cells and CD4^+^Foxp3^−^ Teff cells in different ways; impacting upon the proliferation of CD4^+^Foxp3^−^ Teff cells, but not of CD4^+^Foxp3^+^ Treg cells.

### ICOS deficiency results in increased CD4^+^Foxp3^+^ Treg-cell apoptosis during *H. polygyrus* infection

Given that ICOS was dispensable for Foxp3^+^ Treg-cell proliferation during infection, we asked if the defective Foxp3^+^ Treg-cell responses evident in ICOS^−/−^ mice were due to impaired survival. To test this, we measured the ex vivo percentage of apoptotic Annexin V^+^ CD25^+^CD4^+^ T cells in the MLNs of day 7 *H. polygyrus*-infected WT and ICOS^−/−^ mice. As co-detection of Annexin V and intracellular Foxp3 was not possible, CD25 was used as a surrogate marker for Foxp3^+^ Treg cells. Previous work has demonstrated that CD25 expression accurately reflects Foxp3 expression at this stage of *H. polygyrus* infection 41, however, it should be noted that we cannot exclude the possibility that the CD4^+^CD25*^+^* population included a small proportion of Foxp3^−^ Teff cells. The proportion of CD25^+^ Treg cells undergoing apoptosis in naive ICOS^−/−^ mice was significantly greater than in naïve WT controls, indicating that ICOS contributes to CD25^+^Foxp3^+^ Treg-cell survival under normal homeostatic conditions (Fig. 5C). Upon infection, the percentage of apoptotic Annexin V^+^ CD25^+^ Treg cells increased in both strains, remaining significantly higher in the ICOS^−/−^ mice. A similar pattern of apoptosis was seen within the CD4^+^CD25^−^ Teff-cell compartment (Fig. 5D). Therefore, CD25^+^Foxp3^+^ Treg cells from ICOS^−/−^ mice displayed a higher level of apoptosis under both homeostatic conditions and in response to *H. polygyrus* infection.

### ICOS deficiency specifically impairs Foxp3^+^ Treg-cell, but not Foxp3^−^ Teff-cell, IL-10 production

**Figure 6:**
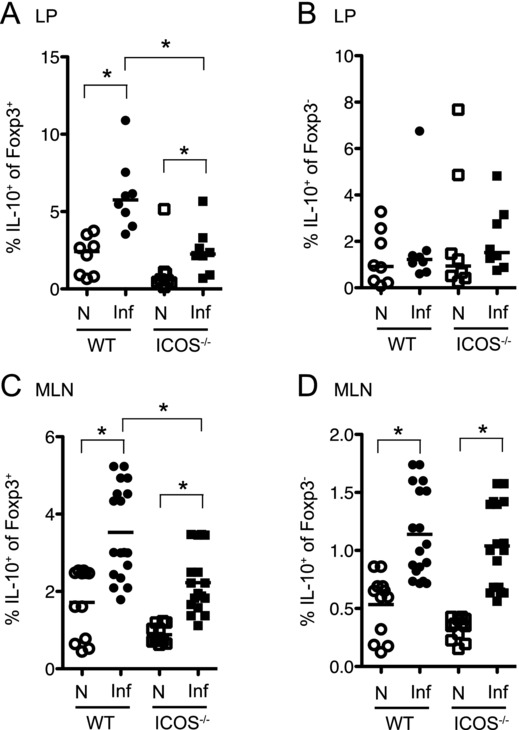
ICOS is required for the production of IL-10 by Foxp3^+^ Treg cells, but not Foxp3^−^ Teff cells. WT (circles) and ICOS^−/−^ (squares) mice were infected with *H. polygyrus* and flow cytometry was used to quantify the intracellular production of IL-10 by (A, C) CD4^+^Foxp3^+^ Treg cells and (B, D) CD4^+^Foxp3^−^ Teff cells isolated from the (A, B) LP and (C, D) MLN at day 7 pi. Data shown are pooled from two (A and B) or three (C and D) independent experiments. Open symbols represent naïve mice and closed symbols represent infected mice. Lines denote mean values. *p < 0.05 (Tukey HSD, ANOVA using combined data from two or three experiments).

To determine whether the functional quality of Foxp3^+^ Treg cells was also impaired in ICOS^−/−^ mice, we tested their ability to produce the regulatory cytokine IL-10. LP lymphocytes and MLN cells were isolated from naïve and *H. polygyrus* infected WT and ICOS^−/−^ mice, and the production of IL-10 by CD4^+^Foxp3^+^ Treg cells and CD4^+^Foxp3^−^ Teff cells was assessed by intracellular staining and flow cytometry at day 7 pi. In the LP of WT mice following *H. polygyrus* infection, elevated numbers of IL-10^+^ cells were only observed in the CD4^+^Foxp3^+^ Treg-cell population (Fig. 6A and B and Supporting Information Fig. 3). In contrast, in the MLNs both CD4^+^Foxp3^+^ and CD4^+^Foxp3^−^ Teff cells increased IL-10 production in response to *H. polygyrus* (Fig. 6C and D). Thus, CD4^+^Foxp3^+^ Treg cells appear to be the predominant T-cell source of IL-10 at the infection site, whereas in the LN IL-10 also emanates from CD4^+^Foxp3^−^ Teff cells. Production of IL-10 by CD4^+^Foxp3^+^ Treg cells was significantly impaired by 60% in the LP and 37% in the MLN in the absence of ICOS (Fig. 6A and C). Interestingly, IL-10 production by MLN Foxp3^−^CD4^+^ Teff cells was unimpaired in the absence of ICOS (Fig. 6D). Together, these data suggest that ICOS is partially required for production of IL-10 by Foxp3^+^ Treg cells, but that it is dispensable for IL-10 production by Foxp3^−^CD4^+^ Teff cells.

## Discussion

Helminth infections are known for their ability to invoke strong immunoregulatory responses. In particular, Foxp3^+^ Treg cells have prominent roles in downregulating protective and pathogenic Th2 responses towards helminths 42. However, little is as yet known about the co-stimulatory environment in vivo which favours the differentiation and expansion of Foxp3^+^ Treg cells during these infections. ICOS is a key co-stimulatory receptor and, alongside its role in promoting CD4^+^ Teff-cell responses, it is becoming clearer that ICOS can influence Foxp3^+^ Treg-cell development and function in autoimmune settings 18,19,21. This study demonstrates that ICOS is partially required for the expansion and long-term maintenance of Foxp3^+^ Treg cells during *H. polygyrus* and *S. mansoni* infections, with the absence of ICOS leading to increased apoptosis of Foxp3^+^ Treg cells. Most notably, Foxp3^+^ ICOS^−/−^ Treg cells were markedly deficient in their ability to produce IL-10, whilst ICOS^−/−^ Foxp3^−^ Teff cells remained IL-10 competent, indicating a selective role for ICOS in promoting Foxp3^+^ Treg-cell derived IL-10.

The numbers of Foxp3^+^ Treg cells in the MLN and spleen during *H. polygyrus* and *S. mansoni* infections were greatly reduced in ICOS^−/−^ mice, showing that the expansion of Foxp3^+^ Treg cells in these settings depends upon ICOS co-stimulation; similar to previously described requirements for ICOS in Foxp3^+^ Treg-cell function in models of tolerance and autoimmunity 18,19,21. This was likely due to a direct impact of ICOS deficiency on Foxp3^+^ Treg cells, as ICOS^−/−^ Foxp3^+^ Treg cells show intrinsic functional defects in a model of Type 1 diabetes 19. However, we cannot rule out the possibility of an indirect effect, as for example, B cells can promote Foxp3^+^ Treg-cell expansion 43 and B-cell function is impaired in ICOS^−/−^ mice 31,44. The expansion of CD4^+^Foxp3^−^ Teff cells was also reduced in the absence of ICOS as observed in other helminth infections 28–31. Interestingly, we found that in contrast to Foxp3^−^ Teff cells where ICOS had an important role promoting their proliferation, ICOS was entirely dispensable for Foxp3^+^ Treg-cell proliferation in vivo. Thus, ICOS regulates the expansion of Foxp3^+^ Treg cells and Foxp3^−^ Teff cells via distinct mechanisms. ICOS is known to aid the survival of T cells and NKT cells 21,45,46 and, in keeping with these studies, we found that in the absence of ICOS the survival of CD4^+^CD25^+^ Treg cells, as well as CD4^+^CD25^−^ Teff cells, was impaired in both naïve and *H. polygyrus*-infected mice. Thus, the reduced expansion and maintenance of ICOS^−/−^ Foxp3^+^ Treg cells during *H. polygyrus* infection is at least partly due to their impaired survival.

Under homeostatic conditions the effect of ICOS deficiency on the Foxp3^+^ Treg-cell population differed depending on immune location. Whilst the proportion of Foxp3^+^ Treg cells was reduced within the LNs of naïve ICOS^−/−^ mice in agreement with previous studies 21,47, there was a dramatically increased percentage of Foxp3^+^ Treg cells within the LP. This was associated with elevated expression of CD103 and CD25 indicating that the Treg cells were in a heightened state of activation. Despite this increased Foxp3^+^ Treg-cell activity in the naïve setting, ICOS^−/−^ mice failed to mount an elevated Foxp3^+^ Treg-cell response within the LP upon *H. polygyrus* infection. The mucosal environment has a propensity for the generation of adaptive Foxp3^+^ Treg cells 37, and *H. polygyrus* secretes a TGF-β mimic capable of inducing Foxp3 expression in naïve T cells 36. In agreement with this, and based on expression of the proposed natural Foxp3^+^ Treg-cell marker Helios 32, the LP Foxp3^+^ Treg cells generated in response to *H. polygyrus* infection of WT mice were all Helios^−^Foxp3^+^ adaptive Treg cells. This indicates that *H. polygyrus* primarily induces an adaptive Foxp3^+^ Treg-cell response at the infection site, bearing in mind that the use of Helios as a natural Foxp3^+^ Treg-cell marker may not be accurate in all immune contexts 48–50. Although studies indicate that ICOS is not required for the induction of adaptive Foxp3^+^ Treg cells in vitro 47, LP Helios^−^Foxp3^+^ Treg cells failed to expand in ICOS^−/−^ mice in response to *H. polygyrus* suggesting that ICOS is required for intestinal adaptive Foxp3^+^ Treg-cell responses during infection.

Alongside diminished expansion, ICOS^−/−^ LP and MLN Foxp3^+^ Treg cells were impaired in their ability to produce IL-10 in response to *H. polygyrus*. Foxp3^+^ Treg-cell-derived IL-10 is important in the regulation of intestinal homeostasis 51, and increased numbers of Foxp3^+^ Treg cells are observed in the colon of mice with a Treg-cell-specific deletion of IL-10 52. Thus, the higher basal levels of Foxp3^+^ Treg cells seen in the LP of naïve ICOS^−/−^ mice may represent an attempt to compensate for a functional deficiency in IL-10. Whilst high ICOS expression is associated with IL-10 in CD4^+^ Teff cells 22,23, in *H. polygyrus* infection the IL-10 impairment was specific to Foxp3^+^ Treg cells. Thus, similar to proliferation where the requirement for ICOS was cell specific, ICOS deficiency had a differential impact upon the effector functions of Foxp3^+^ Treg cells and Teff cells.

ICOS deficiency is ordinarily associated with impaired Th2 cytokine production within secondary lymphoid tissue 28–30. Recent work indicates this may be predominantly due to the loss of ICOS-dependent IL-4 secreting Tfh cells 38–40, and there is evidence that ICOS-ICOSL interactions are not necessary for T-cell IL-4 production 30,31,39. IL-4 competent CD4^+^ T cells commit to the Tfh lineage and enter the follicles between days 6 and 10 of *Leishmania major* infection 39, and within this time frame (day 7 of *H. polygyrus* infection) we found IL-4 production by MLN CD4^+^ T cells was indeed unaffected by ICOS deficiency. Most significantly, the loss of IL-4 protein occurred at later time points following the failure of MLN CXCR5^+^ Tfh cells to expand. Importantly, at the infection site, in the absence of Tfh cells, we found that ICOS deficiency actually led to an increased percentage of CD4^+^ T cells producing IL-4 and IL-13 protein. Thus, in contrast to IL-4-secreting CXCR5^+^ Tfh cells, not only are Th2 effector cell responses efficiently generated in ICOS^−/−^ mice, it appears that ICOS is in fact involved in suppressing Th2 cell effector responses at the infection site. Foxp3^+^ Treg cells accounted for the majority of CD4^+^ T-cell-derived IL-10 within the LP even though Foxp3^+^ Treg-cell functions in *H. polygyrus* infection are reported to be IL-10 independent 15,53. IL-10 and adaptive Foxp3^+^ Treg cells are known to suppress Th2 cytokine production at mucosal surfaces 25,54–56, and so the increased Th2 responses seen within the LP of *H. polygyrus*-infected ICOS^−/−^ mice may be a consequence of functionally impaired Foxp3^+^ Treg cells.

In summary, this study demonstrates that ICOS plays key roles in eliciting Foxp3^+^ Treg-cell responses during infections with the nematode *H. polygyrus* and the trematode *S. mansoni,* both locally at the infection site and systemically within the LN and spleen. In contrast, the regulation of Type 2 immunity towards *H. polygyrus* by ICOS is tissue specific. Within the LN ICOS promotes Type 2 responses being required for the expansion of CD4^+^CXCR5^+^ Tfh cells. However, it is not necessary for Th2 cell priming, and downregulates Th2 cell function at the infection site. Thus, ICOS regulates Type 2 immunity in a tissue-specific manner, and plays a key common role driving Foxp3^+^ Treg-cell expansion and function during distinct helminth infections.

## Materials and methods

### Animals, infections and in vivo BrdU treatment

C57BL/6 and ICOS^−/−^ mice 33 were bred in-house and maintained under specific pathogen-free conditions at the University of Edinburgh. Mice were used at 6–8 weeks of age. All animal work was approved by the University of Edinburgh Ethics Committee (PL02–10) and by the UK Home Office (PPL60/4104), and conducted in accordance with the Animals (Scientific Procedures) Act 1986. Male mice were infected with 200 *H. polygyrus bakeri* L3 larvae by oral gavage. *Biomphalaria glabrata* snails infected with *S. mansoni* were obtained from F. Lewis (Biomedical Research Institute, Rockville, MD). Female mice were infected percutaneously with 70 *S. mansoni* cercariae. To label dividing cells in vivo*,* mice were injected with 1 mg BrdU (Sigma-Aldrich) in PBS i.p. 24 h prior to autopsy.

### Cell purifications and in vitro restimulations

MLNs and spleens were dissociated to obtain a single cell suspension in RPMI 1640 (Invitrogen) supplemented with 100 U/mL penicillin, 100 μg/mL streptomycin, 2 mM l-glutamine and 5% FCS. To isolate LP mononuclear cells, external adipose tissue and peyers patches were removed. The small intestine was opened longitudinally, washed in cold RPMI 1640 (Invitrogen) supplemented with 100 U/mL penicillin, 100 μg/mL streptomycin, 3% FCS and 0.02 M Hepes (Sigma)*,* cut into 1 cm pieces, and washed three times in Wash Buffer (RPMI 1640 supplemented with 100 U/mL penicillin, 100 μg/mL streptomycin, 0.02 M Hepes and 2 mM EDTA (Invitrogen)). The small intestine segments were incubated for 15 min at 37°C in wash buffer supplemented with 3% FCS, 0.16 mg/mL DTT (Sigma) and 5.5 mM EDTA, then washed three times followed by incubation for 30 min at 37°C in RPMI 1640, supplemented with 100 U/mL penicillin, 100 μg/mL streptomycin, 2 mM l-glutamine, 0.02 M Hepes, NEAA (Invitrogen), 1 mM sodium pyruvate (Invitrogen), 0.5 mM β-mercaptoethanol, 0.1 mg/mL liberase TL (Roche) and 0.5 mg/mL DNase I (Sigma). The digested small intestine was passed through 70 and 40 μm filters (BD Biosciences) to obtain a single cell suspension. For measurement of intracellular cytokines, cells were stimulated for 4 h with 0.5 μg/mL PMA and 1 μg/mL ionomycin, with 10 μg/mL Brefeldin A added for the final 2 h (Sigma Aldrich).

### Flow cytometry

The following Abs were used: Alexa Fluor 700-conjugated anti-CD4 (RM4–5, BD Bioscience), allophycocyanin-conjugated anti-Foxp3 (FJK-16s, ebioscience), phycoerythrin-conjugated anti-Helios (22F6, Biolegend), phycoerythrin-conjugated anti-ICOS (7E.17G9, Biolegend), phycoerythrin-conjugated anti-CD25 (PC61 5.3, Invitrogen), phycoerythrin-conjugated anti-IL-4 (11B11, Biolegend), PE-Cy7 conjugated anti-PD-1 (RMP1–30, Biolegend), biotin-conjugated anti-CD103 (M290, BD Bioscience), biotin-conjugated anti-CXCR5 (2G8, BD Bioscience), Alexa Fluor 647-conjugated anti-IL-13 (ebio13A; ebioscience), fluorescein isothiocyanate-conjugated anti-BrdU with DNase (B44, BD Bioscience), allophycocyanin-conjugated Annexin V (BD Bioscience), Pacific Blue-conjugated anti-TCR-β (H57–597, Biolegend). Non-specific binding was blocked with 4 μg of rat IgG per 1 × 10^6^ cells. Intracellular staining for Foxp3 and Helios was performed using a Foxp3-staining buffer kit (eBioscience). For intracellular cytokines, dead cells were excluded using Aqua Dead Cell Stain kit (Molecular probes) and cells were permeabilised using the BD cytofix/cytoperm kit. Annexin V staining was performed as per the manufacturer's instructions (BD Bioscience). Flow cytometry was performed using a FACSCanto 2, or an LSR 2 (BD Biosciences), running FACSDiva software (BD Biosciences). Analysis was performed using Flowjo (Tree star).

### Statistics

Statistical analysis was performed using JMP version 8 (SAS). Parametric analysis of combined data from multiple repeat experiments, or experiments containing more than two groups, was performed using ANOVA, followed by Tukey HSD post-hoc tests. For non-parametric data, the unpaired Mann–Whitney *U* test was used. Figures show means when parametric tests were used, and medians when non-parametric tests were used.

## Abbreviations

LP: lamina propria
pi: post-infection
Teff cell: effector T cell
Tfh cell: T follicular helper cell

## Supplementary material

As a service to our authors and readers, this journal provides supporting information supplied by the authors. Such materials are peer reviewed and may be re-organized for online delivery, but are not copy-edited or typeset. Technical support issues arising from supporting information (other than missing files) should be addressed to the authors.
