# Supplementary material for: ICOS controls Foxp3+ regulatory T-cell expansion, maintenance and IL-10 production during helminth infection
Source: Eur J Immunol. 2013 Jan 14;43(3):705–15. doi: 10.1002/eji.201242794 (PMC3615169; doi:10.1002/eji.201242794)
Supplement: Supplementary file 1 [file eji0043-0705-SD1.pdf]

European Journal of Immunology

Supporting Information
for

DOI 10.1002/eji.201242794

Stephen A. Redpath, Nienke van der Werf, Ana M. Cervera, Andrew S. MacDonald, David Gray, Rick M. Maizels and Matthew D. Taylor

ICOS controls Foxp3^{+} regulatory T-cell expansion, maintenance and IL-10 production during helminth infection

© 2013 WILEY-VCH Verlag GmbH & Co. KGaA, Weinheim

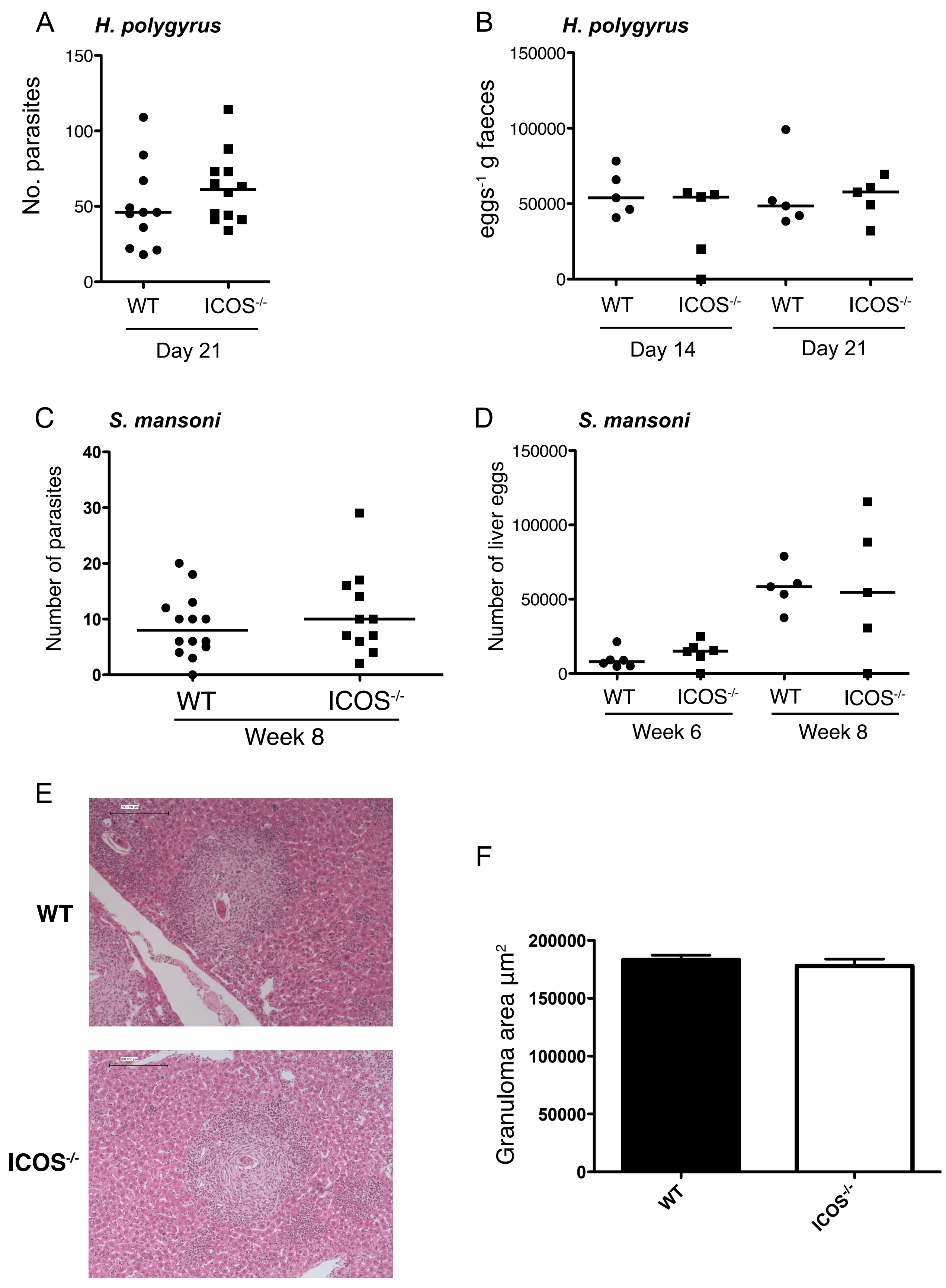

Supplementary Figure 1. ICOS deficiency does not affect susceptibility to *H. polygyrus* or *S. mansoni* infections, or *S. mansoni*-mediated pathology. (A-B) WT and ICOS$^{-/-}$ mice were infected with 200 *H. polygyrus* L3 larvae and the parasite burdens were assessed at d 21 (A), and the number of *H. polygyrus* eggs in the faeces were quantified at d 14 and d 21 (B). (C-F) WT and ICOS$^{-/-}$ mice were infected with 70 *S. mansoni* cercariae and the number of adult parasites quantified at wk 8 of infection (C), and the number of *S. mansoni* eggs in the liver were quantified at wk 6 and wk 8 pi (D). To measure granuloma size liver sections were stained with H & E and the area of immune infiltrate surrounding individual eggs was measured using a compound microscope and Leica analysis software. (E) Representative micrographs of *S. mansoni* egg induced granulomas. Scale bar = 200μm (F) Area in μm^2 of hepatic granulomas. (A-D) Closed circles denote *H. polygyrus* or *S. mansoni* infected WT mice and closed squares denote *H. polygyrus* or *S. mansoni* infected ICOS$^{-/-}$ mice. Data points represent individual mice and bars show median values. (F) Bars depict mean and standard deviation. A & C show pooled data from 3 independent experiments. B shows data from one experiment. D - F show representative data from three independent experiments, two at wk 6, and one at wk 8.

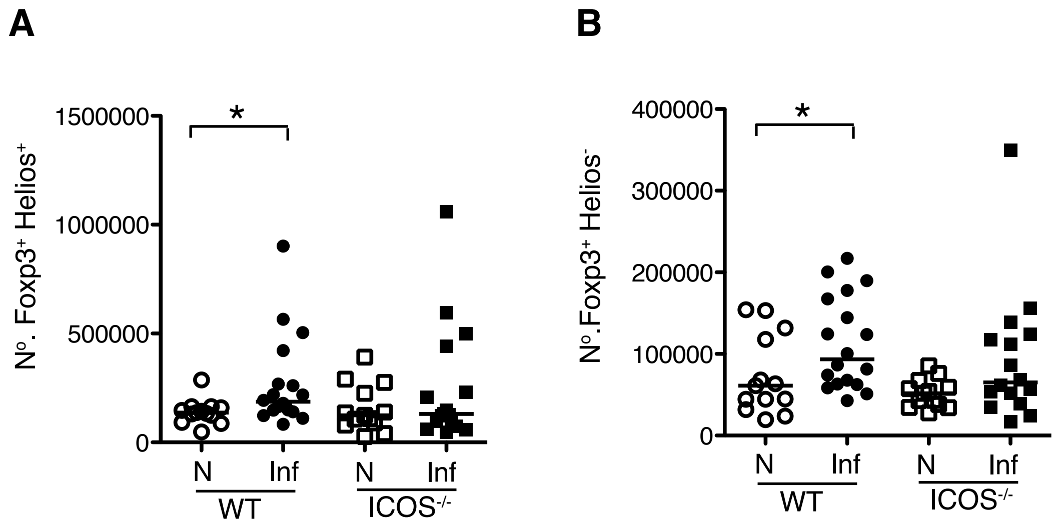

Supplementary Figure 2. *H. polygyrus* infection expands both Helios$^+$ and Helios$^-$ Foxp3$^+$ Tregs in the MLN. WT (circles) and ICOS$^{-/-}$ (squares) mice were infected with *H. polygyrus* and the number of Helios$^+$Foxp3$^+$ (A) and Helios$^-$Foxp3$^+$ Tregs (B) quantified in the MLN at d 7 pi. Open and closed symbols represent naive and infected mice respectively. Panels show combined data from three independent experiments. Lines denote means. * $p < 0.05$ (Tukey's HSD, ANOVA performed on combined data from three independent experiments).

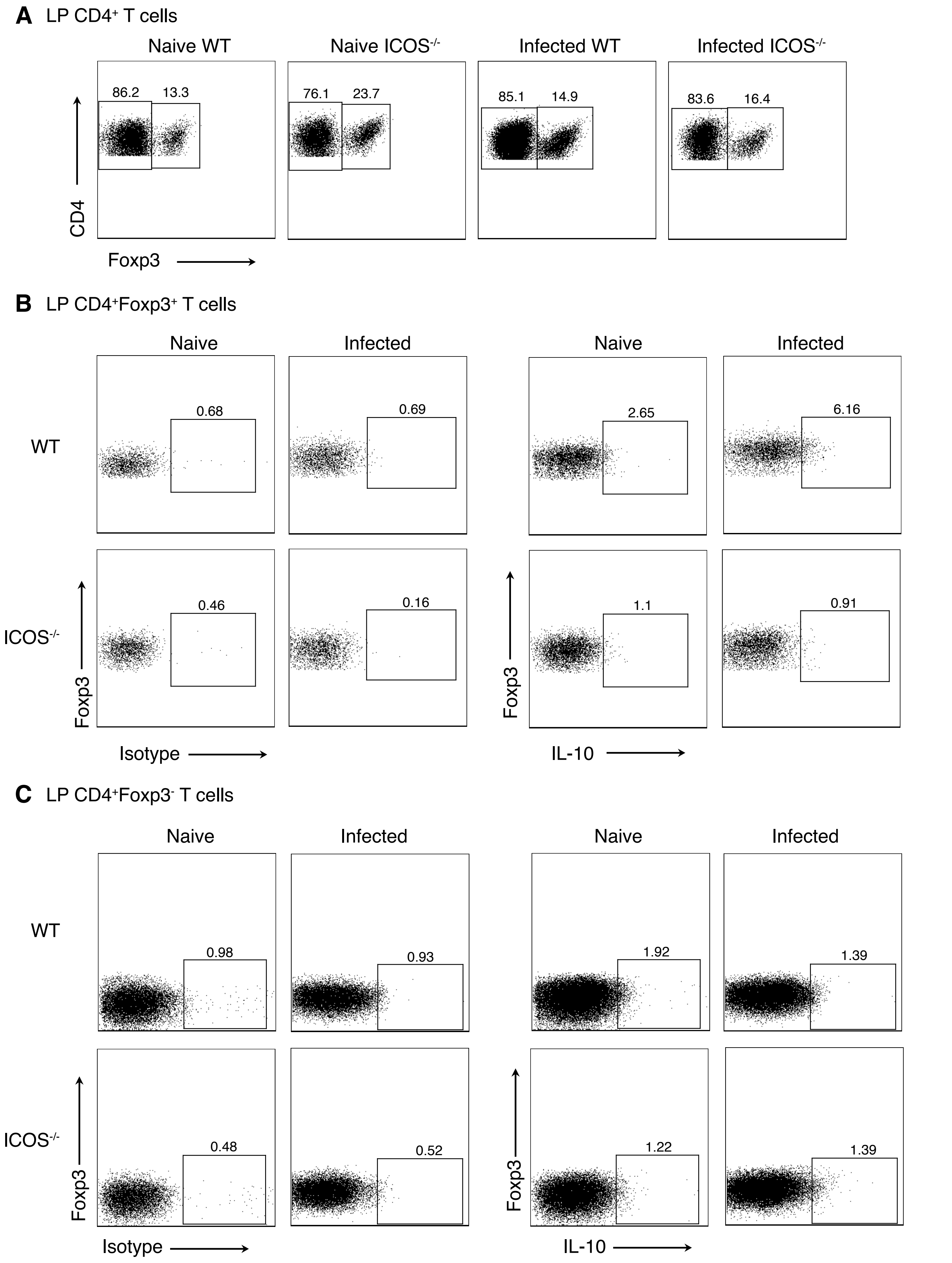

Supplementary Figure 3. Representative FC staining for Foxp3 and IL-10 on LP CD4⁺ T cells. LP cells were isolated from naive and *H. polygyrus* infected mice 7 d pi, stimulated with PMA and ionomycin, and stained for CD4, Foxp3, and IL-10. (A) Gating strategy identifying $CD4^+Foxp3^+$ Tregs and $CD4^+Foxp3^-$ Teff cell populations. (B) Representative staining for Foxp3 versus IL-10 on $CD4^+Foxp3^+$ Tregs. (C) Representative staining Foxp3 versus IL-10 on $CD4^+Foxp3^-$ Teff cells.
